# A Comprehensive Evaluation of Feasibility and Acceptability of a Nurse-Managed Health Clinic for Homeless and Working Poor Populations: A 3-Year Study

**DOI:** 10.3390/nursrep15120412

**Published:** 2025-11-21

**Authors:** Teresa M. McIntyre, Shainy B. Varghese, William Pat Taylor

**Affiliations:** 1 Andy and Barbara Gessner College of Nursing, University of Houston, 14000 University Boulevard, Sugarland, TX 77479, USA; sbvarghese2@uh.edu; 2 Texas Institute for Measurement, Evaluation, and Statistics, University of Houston, Houston, TX 77204, USA; pat.taylor@times.uh.edu

**Keywords:** nursing, homelessness, primary care, nurse-managed clinics, retention, patient satisfaction, Nurse Practitioners, social determinants of health

## Abstract

**Background/Objectives:** Homeless populations have higher rates of chronic illness and mortality than more advantaged peers but have low primary care engagement. Nurse-managed clinics emerged as a possible solution to increase healthcare access for marginalized populations. This paper presents a comprehensive evaluation of feasibility (conceptualized as patient recruitment and retention) and acceptability (conceptualized as patient satisfaction) of a nurse-managed primary care clinic tailored to people experiencing homelessness and poverty. **Methods:** This is a three-year retrospective chart review study of the clinic’s services, patient characteristics, and patient satisfaction. All adult patients for the three-year period were included (*N* = 514). Feasibility was measured by the number of unique patients seen and visits completed, ratio of completed to scheduled visits, and number of returning patients. Acceptability was measured by a 19-item Likert format (1–5) patient satisfaction survey. Patient characteristics were captured from intake forms. **Results:** Most patients were male, African American or White, and non-Hispanic. Regarding social determinants of health (SDOH), most patients did not have college education, were unemployed or unable to work, experienced homelessness, had no primary care provider, and no health insurance. Over three years, 1972 visits were scheduled and 1372 (69.6%) completed. A total of 514 patients were seen (37.5% of all visits), with 858 follow-up visits (62.5%). Returning patients (≥2 visits) totaled 59.1%. Yearly data shows steady growth in recruitment and retention. Patient satisfaction with facets of care (access, communication, interpersonal relations) was very high (*M*range = 4.63–4.69), including with Nurse Practitioner care, as was global satisfaction (*M* = 4.71; *SD* = 0.61; 76.3% very satisfied). **Conclusions:** Results indicate that a homeless-tailored nurse-managed clinic can recruit and retain homeless and working poor patients (feasibility), with high patient satisfaction with its services and staff (acceptability), independently of patient demographics or SDOH. Challenges related to retention deserve further study as well as the impact of services on the continuity of care, health, and well-being.

## 1. Introduction

Homelessness, a lack of fixed or stable housing, has increased globally and in the U.S., constituting a complex public health challenge with significant social, economic, and health consequences. The 2024 Annual Report on Homelessness to the U.S. Congress indicates that 771,480 people (23 in 10,000) experienced homelessness in one night in 2024, the highest number ever recorded [1], and also highlights a 32.9% increase in homelessness from 2020 to 2024. The National Alliance to End Homelessness reported that, in 2024, first-time homelessness increased by 23% compared with 2019 [2]. This trend is worrisome since experiencing homelessness is associated with physical, mental, and social burdens that synergistically result in increased morbidity and mortality [3,4]. In the U.S., homelessness is associated with up to 10 times higher mortality rate than housed individuals [5]. Homelessness is also related to higher rates of infectious disease, chronic disease, serious mental illness, substance abuse, and suicide than housed populations, all contributing to higher mortality and morbidity, and lower quality of life [3,6].

Despite increased and unmet health needs, unhoused populations have lower access to care, especially primary care, lower engagement with health services, and poor treatment adherence [7,8,9]. These populations face extreme social and structural disadvantages, or social determinants of health (SDOH), which coalesce in decreased access to primary care services that provide much-needed preventive care, chronic disease management, and continuity of care [10]. Instead, homeless populations rely on acute services for care, showing increased emergency room use and hospitalizations, often for preventable conditions, thus overloading the healthcare system [11,12]. Barriers to healthcare access include poor health, high stress, unhealthy and dangerous environments, food insecurity, lack of health insurance, unemployment, poor education and health literacy, and mistrust of the healthcare system [3,10]. There is agreement that responding to the healthcare needs of homeless populations is urgent and requires innovative models of care and community partnerships involving healthcare, social service, and education agencies [13,14].

Different models of care have been implemented to increase access to care among homeless populations, including medical student-run clinics, mobile clinics, and street medicine, which bring care to spaces where people experiencing homelessness live, and placing homeless-tailored primary care clinics with emergency room departments as patient-centered medical homes [15,16,17]. Studies have found that these initiatives have positive outcomes for people experiencing homelessness by increasing access to care and reducing burden on the healthcare system in emergency visits and hospitalizations [15,16]. However, challenges have also been noted such as a need of linkage to formal primary care services, low acceptability of primary care services in a hospital environment by clinicians/staff, services configuration, and continuity of care [8,15,17]. While a wide range of services were provided by these initiatives, there is consensus that access to formal primary care is essential for reducing healthcare disparities among those experiencing homelessness [18].

Nurse-managed health clinics (NMHCs) have emerged as a possible avenue to increase primary healthcare access for marginalized populations [19,20]. They are led by Advanced Practice Registered Nurses (APRNs) or Nurse Practitioners (NPs) who provide quality and affordable healthcare to vulnerable populations [21]. NPs are APRNs who completed a master’s or doctoral-level education and obtained board certification. They are licensed and authorized to evaluate patients, diagnose patient problems, order and interpret diagnostic tests, and initiate and manage treatments, including prescribing medications and controlled substances under the licensure authority of a state board of nursing [22,23,24]. NPs also serve as healthcare researchers, interdisciplinary consultants, and patient advocates, and NPs can work in diverse healthcare settings including clinics, hospitals, private physician and NP practice, nursing homes, schools, nurse-managed clinics, homeless clinics, and home health [25]. NPs in primary care have shown to increase access to care with increased patient satisfaction, also addressing provider shortage [21,26,27]. Nurse-led clinics are less costly compared to physician-led care, and nurses can achieve similar or better outcomes than physicians in terms of patient retention and improved health on a broad range of conditions, especially NPs [27,28,29]. Their role in expanding primary care capacity was identified in the Patient Protection and Affordable Care Act (2010) that identified NMHCs as a healthcare delivery model that can improve healthcare accessibility for low-income and minority populations [30]. Despite their increasing proliferation and recognition, there is lack of research on NMHCs’ feasibility and acceptability as well as care processes and outcomes across settings of practice, especially with underserved populations such as those experiencing homelessness or poverty [8,31].

This paper presents a comprehensive evaluation of feasibility (focused on patient recruitment and retention) and acceptability (conceptualized as patient satisfaction) of a nurse-managed primary care clinic tailored to people experiencing homelessness and poverty. Feasibility and acceptability data will contribute to much needed evidence on the viability of nurse-led clinics for homeless and working poor populations and the acceptability of NP care. The association between demographic characteristics and SDOH and patient satisfaction were explored. This report is part of an on-going larger study on healthcare needs, health and well-being, and patients’ perceptions of services at an NMHC for homeless and working poor populations [32].

## 2. Materials and Methods

### 2.1. Setting

This NMHC was established in midtown Houston, TX, in 2021 under the leadership of the University of Houston (UH) Andy and Barbara Gessner College of Nursing, at a strategically located facility of a church, next to an interfaith non-profit providing food, clothing, and other basic needs to more than 5000 homeless and working poor people, weekly. The clinic follows the NASEM recommendations for primary care services with marginalized populations [21], building on a collaboration between a College of Nursing, community-based social agencies, and faith-based organizations serving the homeless population [32]. This NMHC shifts the paradigm from patients having to travel for services to bringing services to them, thus building on previously built relationships and trust and facilitating access to care. These services are urgent because, similar to other U.S. cities, there has been an increase in Houston’s homeless population, with the 2025 annual point-in-time count recording 3325 homeless residents (sheltered and unsheltered) in three counties, most in Harris County (90%), where the clinic resides [33].

### 2.2. Intervention: The UH College of Nursing Health Clinic

The UH College of Nursing Health Clinic (UHCON-HC) is a university-based NMHC at a service location, with the mission to provide primary healthcare for those experiencing homelessness and the working poor. It is important to note that most patients of the NMHC are referred by non-profits that have vetted their patients for eligibility to services based on their income, including meeting the criteria of being financially indigent per 200% or below the FPL criteria, which is consistent with the designation of economically disadvantaged. The clinic provides comprehensive direct primary care in a flexible, client-centered model and has virtual care capabilities. The UHCON-HC is staffed by UH faculty (NPs) and certified nurses, a collaborating physician, a medical assistant, a receptionist, and a security officer. The UHCON-HC is directed by an NP faculty and supported by administrative and technical staff from UH. Staff are vetted for prior experience with homeless and other underserved populations. The UHCON-HC also serves as an interprofessional training site for nursing, optometry, and dental and medical students, contributing to a future workforce that is prepared to address the unique healthcare needs of homeless and working poor populations.

Nurse-Led Model of Care (NLMC) is an evidence-based model that has emerged as a high-quality cost-effective model of healthcare delivery [34]. The NLMC is led by APRNs, and the scope is broad, encompassing patient-centered care aiming to improve outcomes and address healthcare access gaps and primary care provider shortage. UHCON-HC follows a Nurse-Led Model of Care in Texas, where the scope of NP practice is restricted and requires a collaborating physician for supervision. The APRNs have signed the APRN–physician collaborative agreement. NPs at the clinic hold master’s and/or doctoral degrees in nursing and are board-certified.

The UHCON-HC offers a variety of primary care services including managing acute and chronic care conditions (e.g., hypertension, diabetes, obesity), preventive visits, well-woman exams, immunizations, health education, and specialist referrals as needed. In addition, NPs at the clinic offer immunizations, which is vital in preventing infectious disease and promoting public health. Another significant aspect of care offered at UHCON-HC is the availability of on-site medication and laboratory tests. Patients benefit from these services at no cost, removing financial barriers that might prevent them from seeking necessary healthcare. This includes routine blood tests, screenings, and other point-of-care testing such as urine analysis, COVID-19, flu testing, etc. Another service offered at the clinic is TB skin test, a test that is crucial for the early detection of TB, especially in the homeless and underserved communities due to their exposure. Positive tests are reported to the public health department for further management and follow-up. This service contributes to a greater public health strategy aimed at protecting communities.

The clinic is open two days a week, from 8:30 am to 15:30 pm, and serves, on average, eight patients per day. Optometry screenings and dental van services are available monthly via university partnerships. Direct and virtual mental health services will be added in 2025, with the addition of a psychiatric nurse practitioner. The UHCON-HC also conducts health fairs and screenings at shelter and food delivery locations at the request of its community partners. The clinic works in collaboration with an Advisory Board, with representatives from its stakeholders who support fundraising and clinic development. The UHCON-HC is grant-funded and receives in-kind support from the UH and a church partner.

### 2.3. Study Design and Measures

This is a three-year retrospective chart review study of the clinic’s services, patient characteristics, including SDOH, and patient satisfaction. It is an exploratory, descriptive, and correlational study [35]. Data was captured from electronic patient records and paper forms for the first three years of services, from September 2021 to August 2024. All adult patients for the three-year period were included in this study (*N* = 514).

The review of clients’ charts was for limited data pertaining to intake information and routine survey assessments collected as part of the client’s first visit. Data was de-identified to protect the client’s confidentiality, and other precautions were taken in this regard (e.g., data encryption, secure file transfer systems). Patient demographic characteristics (age, gender, race, ethnicity, and marital status) and SDOH (education, employment, housing, transportation, and accessibility to care, including ER use, and having health insurance) were retrieved from clients’ intake form [36]. Disability and veteran status, as well as the clients’ reported top health issues, were also captured.

Feasibility (conceptualized as patient recruitment and retention) was measured by the number of unique patients seen, visits completed for the three years of clinic operations, ratio of completed to scheduled visits, and the number of returning patients (retention). Yearly data on feasibility were also examined. Retention was also measured by number of return visits per patient and the number of canceled or missed appointments.

Acceptability was conceptualized as patient satisfaction, which is compatible with Bowen et al.’s definition of acceptability [37] and with the definition of patient satisfaction as a reaction to, or outcome of, an interaction between patients and healthcare providers [38]. Acceptability (conceptualized as patient satisfaction) was measured by a 19-item Likert scale format (1 = Very dissatisfied to 5 = Very satisfied) patient satisfaction survey (Patient Satisfaction Questionnaire-Brief, PSQ-B, McIntyre, 2021) [39]. Items 1–17 assess three rationally derived dimensions of patient satisfaction (access to care: 7 items; communication with providers/information: 4 items; and patient-provider interpersonal relations: 6 items), item 18 refers to team communication/coordination, and item 19 captures global satisfaction. Five of the 19 items pertain to satisfaction with NP care. For example, Item 2 (Access) states, “Regarding access to medical care when needed, I am:…”), Item 9 (Communication/Information), “Regarding the information that the Nurse Practitioner at this Clinic gives me about my disease or treatment, I am: …”, and Item 16 (Interpersonal relations)” is “Regarding the emotional support that the Nurse Practitioner gives patients at this Clinic, I am: …”. Item 18 (Team coordination) reads, “Regarding communication/co-ordination when I’m being treated by more than one health professional at the same time, I am: …”, and Item 19 (Global satisfaction) states, “In general, my evaluation of the health care that I receive at this Clinic is: …”. An additional open question offers patients the opportunity to give suggestions for improvement: “What do you think would help improve the services offered by this Clinic?” Cronbach’s alpha reliability coefficient for the PSQ-B (18 items) in this sample is 0.99, indicating very good internal consistency reliability, with corrected item–total correlations ranging from 0.86 to 0.97. Cronbach’s alpha reliability for the rationally derived dimensions was 0.98 for Access, Communication/Information, and Interpersonal Relations, with item–total correlations for the scales varying between 0.87 and 0.97. Construct validity of the 18 items was examined using exploratory factor analysis, with principal component analysis producing one factor with eigen value greater than 1, explaining 89.34% of the variance, with the item factor loadings ranging from 0.87 to 0.97. Therefore, a factor score for the 18 items was produced for analyses and labeled “PSQ-B Total Scale” (see Section 3.3).

### 2.4. Analyses

The IBM SPSS statistical package, version 29.0 and SAS version 9.4, were used to conduct analyses. Data analyses included descriptive statistics (e.g., frequency, *M*, and *SD*) to characterize the population served, the clinic’s services and retention (feasibility), and patient satisfaction (acceptability). Given the uneven number of items per scale in the PSQ-B, average scores were computed to facilitate scale score comparison, and with the total average score (items 1–18). One-Way ANOVAs were used to examine yearly changes in patient satisfaction. Bonferroni post hoc tests were conducted to test the significance of pairwise clinic year comparisons. Linear regressions were computed to study the relation between demographic characteristics (gender, age, race, and ethnicity) and SDOH (education, employment, homelessness, access to care-primary care, and health insurance) and patient satisfaction. Separate models were produced for demographic and SDOH factors and for each dependent variable. Due to the small *n* in some variable categories (e.g., “Other” for gender, “Asian” for race), these variables were recoded as follows: Gender (Male, Female); Race (Black/African American, White, Other races); Ethnicity (Hispanic, Not Hispanic or Latino); Education (Less than high school, High school, Some college, BS or greater); Employment (Unemployed or retired, Employed, Unable to work); Age, homelessness, access to primary care, and insurance were not recoded, and marital status was not included in the analyses due to the distribution of the variable as predominantly single. For descriptive statistics (frequencies, percentages, mean, standard deviation), participants who omitted a question were not included in the computations of the statistics. For factor analysis, missing data was handled with listwise deletion to provide a uniform data set. Missing data for regression analyses was handled with pairwise deletion. For all analyses, alpha level (α) was set at 0.05.

## 3. Results

### 3.1. Patient Characteristics and Social Determinants of Health

Table 1 describes the demographic characteristics of patients attending UHCON-HC over three years. Regarding demographic profile, most patients were male (70.8%), and the average age was 49.03 years (*SD* = 13.34), ranging from 18 to 80 years old. Most were African American (57.2%), followed by White (37.8%), 5% being from other races (Asian, American Indian/Alaskan Native, Native Hawaiian/Other Pacific Islander, Arab/Middle Eastern); most were not of Hispanic or Latino ethnicity (67.5%), 18.7% were Hispanic or Latino, and 13.8% were from unknown ethnicity. Regarding marital status, the majority were single (68.8%), followed by separated/divorced (17.6%), married/living with partner (7.9%), widowed (4%), or other status (1.7%).

Table 1 also lists the patients’ SDOH characteristics. Most patients had high school education (26.6%) or a GED (17%), and 16.6% had less than high school; a total of 23.7% had some college education, 7.6% completed a Bachelor’s degree, and 7.1% had an Associate degree, with 1.5% having Master’s or Doctorate degrees. Most were unemployed (53.6%) or unable to work (22.9%), 18.4% worked full- or part-time, and 5% were retired. Forty-two percent reported disability, and 9% were veterans. Most of the patients (73.5%) reported some degree of homelessness, 37.8% indicating they were homeless most of the time, 7.5% often, and 28.3% sometimes. The majority reported living in a shelter (28.5%) or outdoors (23.5%), some in an apartment or house (28.3%), with friends or family (6.4%), transitional housing or group home (12.1%), and 1.1% in other accommodations (hotel/motel or other). Regarding access to care, 82.3% of respondents reported they did not have a primary care provider, and when inquired about whether they were seeing any provider for their health issues, 82.3% also answered “no”. When asked “Have you visited the ER recently?”, 32.7% answered “yes”. Regarding having any type of health insurance, 60.5% reported no health insurance. Access to care was also examined in terms of availability of transportation: “How do you get from one place to another?” (choosing all that apply). Most patients reported using Metro Bus (59.7%) or Metro Train (47.7%), followed by walking (39.7%), using own vehicle (9.5%), friends’ transportation (4.3%), or bicycle (3.1%).

The predominant self-reported health issues were the following: High blood pressure (36.8%), emotional problems (27.6%), smoked/used illicit drugs (18.1%), high cholesterol (15.2%), diabetes (14.9%), asthma (12.3%), obesity (9.7%), and sexually transmitted diseases (5.5%).

### 3.2. Feasibility

Table 2 presents the feasibility data on the UHCON-HC services, including the number of scheduled, completed, canceled, and “no show” appointments over the three years and by year, and type of appointments (new visits and follow-up visits). Over three years, the UHCON-HC scheduled 1972 visits, and 1372 (69.6%) were completed. A total of 514 (37.5%) unique patients were seen, and 858 follow-up visits were completed (62.5%). Returning patients (≥2 visits) totaled 59.1%, with 40.1% of these returning for 2 to 5 visits and 19% for more than 5. The mean number of visits per patient was 2.84 (*SD* = 3.67), with a range from 1 to 28 visits. The ratio of completed/scheduled visits for the three years was 69.6%.

Yearly data reflects the growth of the clinic over the three years of longevity (Table 2), with 181 completed visits in Year 1 (Y1), 465 in Year 2 (Y2) and 726 in Year 3 (Y3). The growth from Y1 to Y2 by 2.57 times is more than the doubling of visits expected with the increase in services from 1 day in Y1 to 2 days in Y2. The number of visits increased 1.56 times from Y2 to Y3. In terms of recruitment, the clinic recruited 94 new patients in Y1, 163 in Y2, and 257 in Y3, reflecting a 1.73 times growth from Y1 to Y2 and a 1.58 times growth from Y2 to Y3. There is a shift in the distribution of visits between new and follow-up visits from Y1 (51.9% new patient visits) to Y2 (35.1%) and Y3 (35.4%), reflecting an increase in follow-up or returning visits in Years 2 and 3. Returning patients (≥2 visits) made up 49.2% of all visits in Y1, 62.2% in Y2, and 59.6% in Y3, also showing increased retention in Years 2 and 3. The visit completion rate increased for Y3 relative to Y1 (67.5%) and Y2 (67.3%).

### 3.3. Acceptability

The PSQ-B survey overall response rate (all items answered) was 77% (*n* = 396), with similar response rates for the different facets of satisfaction, ranging from 73% (Team coordination) to 77% (Access). Table 3 presents descriptive statistics on patient satisfaction with the facets of care (Access, Communication/Information, Interpersonal Relations, and Team Coordination) and global satisfaction. The mean on the 18 PSQ-B items was very high (*M* = 4.66; *SD* = 0.62), as was patient satisfaction with facets of care (*M*range = 4.63–4.69 on the 1–5 scale), including with Team Coordination (*M* = 4.68; *SD* = 0.63). The items with highest patient satisfaction pertained to Interpersonal Relations, namely, item 14, “Regarding, the interest in the patient shown by the care team at this Clinic…”, and item 16, “Regarding, the emotional support that the Nurse Practitioner gives patients at this Clinic…”, with 77% and 76% of patients being very satisfied, respectively. The other four items addressing NP care, showed equally high patient satisfaction (range “Very satisfied” = 72–76%). Global satisfaction with care was very high (*M* = 4.71; *SD* = 0.61), with 76.3% of patients reporting being very satisfied with care at the UHCON-HC.

ANOVA results show significant changes in patient satisfaction over the three years of services for facets of care, total scale, and global satisfaction (*p* range: 0.016 to 0.008). Post hoc comparisons indicate stability of yearly patient satisfaction across all facets of care and global satisfaction from Y1 to Y2 (all *p* > 0.05). Although patient satisfaction remains very high, post hoc comparisons indicate there are significant yearly changes from Y2 to Y3 for all PSQ-B scores (*p* range: 0.017 to 0.008), showing Y3 satisfaction is significantly lower than Y2.

Responses to the open question on the PSQ-B Item 20, “What do you think would help improve the services offered by this Clinic?”, are consistent with the positive evaluation on the PSQ-B survey and did not seem to vary yearly. Although we did not conduct a formal qualitative analysis, we present some examples reflecting the positive evaluation of care as well as suggestions for improvement. The following examples reflect acceptability of the UHCON-HC care: “Clinic was very helpful. Don’t change a thing”; “Nothing is lacking. Great people. Great service.”; “The clinic is well organized and perfect. Very well managed.”; “I received a lot more than I anticipated in terms of help and assistance.”; and “Very friendly and respectful environment.”. Examples of suggestions for improvement are “Have a dental van come once a month and an eye doctor.”; “Longer hours and more days.”; “Warm doughnuts and coffee.”; “Make information more public for more homeless so that they can have access to this health clinic.”, and “More staff with the same hospitality.”.

Regression results for the relation of demographic variables (age, gender, race, and ethnicity) with patient satisfaction scales, PSQ-B Total Scale, and PSQ-B Global Satisfaction did not reach significance (all *p* > 0.05). All regression models for SDOH’s (education, employment, homelessness, insurance, and primary care access status) relation with patient satisfaction scales, total scale, and global satisfaction were also not significant (all *p* > 0.05).

## 4. Discussion

The UHCON-HC was established to address the primary care needs of homeless and working poor populations in midtown Houston. Results on its patients’ demographic characteristics indicate an overrepresentation of males, African American, and single individuals, which aligns with national statistics on people experiencing homelessness in the U.S. [1]. About three quarters of the clinic’s patients reported some degree of homelessness, suggesting that the clinic is recruiting the patients it is intended to serve.

Homelessness is a key SDOH and a barrier to accessing primary care, with the UHCON-HC aiming to address this need. Other SDOH identified for most clinic patients were lack of employment, lack of access to a primary care provider, no health insurance, and lack of independent transportation—barriers to care well documented in the literature that contribute to health inequities among these vulnerable populations [3,10]. The top health issues reported by the clinic’s patients (e.g., hypertension, emotional problems, substance abuse, and high cholesterol) are in line with previous findings [40,41]. Most of these health issues are chronic problems that are amenable to effective treatment or preventable with early treatment, with primary care access being essential [18].

The UHCON-HC implemented an innovative nurse-managed model of care supported by strong academic–community partnerships. Results support its feasibility in terms of patient recruitment, sustainability, and growth of its client population over the three years of services. The yearly growth data, with a relatively slow start in recruitment at Y1, illustrates the challenges of gaining the trust of these vulnerable populations. A well-documented challenge in providing services to homeless and other vulnerable populations is retention, with clients presenting high rates of treatment recidivism due to social and structural barriers such as lack of housing and transportation [42]. Results show that the UHCON-HC had a high rate of return patients, with over half of patients returning for two or more visits, with the ratio of completed to scheduled visits being very high. These results are impressive given the transient nature of this population and the barriers already mentioned, although consistent with previous findings of higher return visits in services provided by nurses than doctors [29].

Acceptability of the clinic’s services as measured by patient satisfaction was very high, with over two thirds of patients reporting very high satisfaction with the overall care and facets of care such as accessibility of the clinic, information given to them about treatment and lifestyle recommendations, respect, emotional support and interest of staff, and team coordination. All items referring to NP care were evaluated with high satisfaction. The responses to the open question about improvements needed yielded similar positive expressions of appreciation for services and staff, and the climate of respect and friendliness. These data replicate previous findings that nurse-managed primary care improves patients’ experience of care and care outcomes [26]. Results showed no significant relation of the NMHC client’s demographic characteristics or SDOH with facets or overall patient satisfaction, suggesting that patients are satisfied with the care received and NP care independently of their age, race, ethnicity or gender, education, employment status, degree of homelessness, and history of accessibility to insurance or a primary care provider. Yearly results showed a slight but statistically significant decrease in patient satisfaction from Y2 to Y3, which needs to be examined. Results may be attributable to changes in patient characteristics from Y2 to Y3. This hypothesis needs further examination to better understand what is driving this change. Also, examining Y4 patient satisfaction data will clarify whether this trend towards reduced patient satisfaction with care continues and needs to be addressed.

By collecting data on patients’ SDOH and their health needs, the clinic made systematic adjustments that facilitated recruitment, growth, and retention. For example, from Y1 to Y2, the clinic increased services to two days a week to increase continuity of care and meet patient demand. The clinic also added snacks and bottled water, given the prevalent food insecurity and dehydration among this population. In Y2 and Y3, in response to patients, the clinic added regular dental van services through a second academic partner, and optometry screenings, and exams. Other patient-oriented services that were meant to overcome access barriers and improve adherence were free medications on site and basic laboratory tests; a partnership with another non-profit providing housing ensured free bus passes to receive basic health screenings at the clinic. More recently, the clinic ensured funding to open much-needed mental health services. Tailored primary care with integrated services is the gold standard in caring for vulnerable populations, including those experiencing homelessness [15]. Another key component is to address the intersection of health and social needs, which the clinic is performing via strategic partnerships with other agencies such as housing and food distribution partners [18].

Over the three years, we learned that sustaining and expanding NMHCs requires more than clinical excellence. Establishing relationships with organizations with similar vision was very helpful in securing funding. In addition to financial, operational, and infrastructure support, community engagement and support are other critical resources. Establishing and operating the clinic has provided valuable lessons. The collaboration of the University, faith-based organization, and community partners are very much needed to make the clinic possible.

Despite the promising feasibility and acceptability results, there are some challenges concerning the UHCON-HC services. Building trust in this population takes significant time and effort, and the increasing number of patients and follow-up appointments attest that trust is being established. Consistency in patient numbers is challenging due to the population’s transient nature. Another challenge is reaching out to patients without reliable communication devices. The clinic is open only two days per week, which limits continuity of care. This limitation is tied to several factors, including staff and NP supply, and funding availability. The clinic director and the NPs are faculty supplied by the Gessner College of Nursing, while registered nurses and other staff are hired, but the competitiveness of the healthcare market makes staff retention a challenge. The clinic relies on philanthropic grants and in-kind support to fund its operations. Despite its success, this business model is becoming more difficult to sustain given recent cuts in federal and state funding for this type of program, which increases competitiveness for philanthropic funding. The clinic and its Advisory Board are examining innovative ways to address these challenges.

A core challenge to nurse-led clinics such as the NMHC are NP scope-of-practice regulations that limit the extent of practice of NPs, as mentioned in Section 2.2. NP practice authority varies by state, and it is categorized as full practice authority, without physician supervision, reduced practice authority where NPs are required to have a collaborative agreement with physician, and restricted practice authority where NPs work under physician supervision. Twenty-seven states have full practice authority, twelve states have reduced practice authority, and eleven states have restricted practice authority [43]. Since Texas is a state with restricted practice authority, NPs have limited autonomy in patient care and face many administrative and legal barriers. The restrictive practice prevents nurses from starting NMHCs, and this limits patient access in underserved areas. In addition, the supervisory agreement also becomes a financial obligation to NPs, and this adds to the operational cost of NP practice. Studies show that states with full practice authority have better overall health, improved access to primary care, higher quality of care, and lower healthcare cost [43]. The state of Texas is classified as “restricted practice” with other eleven states (e.g., Oklahoma, Georgia, California) due to restricting practice in at least one of four domains: (1) evaluate patients, (2) diagnose patient problems, (3) order and interpret diagnostic tests, and (4) initiate and manage treatments including prescribing medications and controlled substances under the licensure authority of a state board of nursing [22,24]. In the case of the UHCON-HC, physician supervision and supervisory agreements are required for NP practice.

The feasibility and acceptability results of this study may be of interest to clinics in states with similar practice regulations to Texas, but may differ from other states, such as neighboring New Mexico (full practice state) or Louisiana (reduced practice state). More research is needed on the impact of these regulation barriers on the feasibility and acceptability of nurse-led clinics.

## 5. Study Limitations

This study carries limitations worth noting such as potential selection bias [44]. The study was conducted in a specific NMHC (single-site), inner-city location, and with specific underserved populations (homeless or working poor), which limits the generalizability of findings. Given the similarity of clients’ demographic and SDOH characteristics to national data on homeless populations [1], study results may be relevant to similar clinics and locations. However, our findings may not translate to rural areas, other underserved communities, or different healthcare contexts. More diverse multi-site studies would aid in determining a broader applicability of the nurse-managed clinic model of care. A strength of this study was its comprehensive evaluation of patient satisfaction, including specific aspects of NP care. A potential limitation is that the patient satisfaction survey data was not anonymous due to the need to link survey and patient chart data, which may have resulted in an overly positive evaluation of care reflected in the low variance in responses. This positive response bias may represent an attempt to please the care team and create a good impression (social desirability bias), more likely in a population that is vulnerable and does not trust the system [45]. Introducing some anonymous reporting would be desirable in future NMHC assessments. Although representativeness of the patient sample in terms of patient satisfaction is good (75% or more respondents), given that about one quarter of patients did not respond to the patient satisfaction survey, another limitation is possible self-selection bias, with patients who did not participate in the survey possibly being more negative in their evaluations of patient care [44]. It would be relevant to investigate the reasons for non-response, with lack of anonymity being a possible factor. Assessments of patient satisfaction were made on the client’s first visit; longitudinal data could help determine whether the clients’ experience of care changes with continued care. Multiple assessments of patient satisfaction are the gold standard in patient satisfaction studies to capture changes in acceptability over time and are recommended for prospective studies [37]. This study focused only on the patients’ perceptions of care. Acceptability by nursing staff and students in training needs to be investigated since patients’ experiences and their adherence outcomes are related to stigma and dehumanization by health professionals [42]. Finally, examining patient health status by incorporating objective clinical outcomes (e.g., blood pressure control, HbA1c levels, preventive care uptake), in addition to services rendered and patient-reported satisfaction, would enhance the evaluation of the NMHC model of care.

## 6. Conclusions

This study contributed to the growing evidence base supporting the viability of NMHCs to increase access to primary care among vulnerable populations and help address physician shortages. The results support the feasibility of a homeless-tailored NMHC and the high acceptability of its services and staff, especially NP care, with this approach deserving replication in similar settings. The clinic attributes its success to the NPs practicing to the full extent of their education and training, although within regulatory restrictions, the support of the academic–community partners, and the continuous monitoring of patient experience and needs. The UHCON-HC adopts a holistic approach and understanding of the needs of its vulnerable populations, which facilitates healthcare access through building client trust [46]. The high acceptability indicates that the longitudinal and relationship-based nature of nurse-managed care may help to overcome the stigma and distrust that vulnerable populations experience vis-a-vis the healthcare system [42], increasing the probability of retention, treatment adherence, and the effectiveness of healthcare.

Although the literature consistently confirms that nurse-led models of care are cost-effective and enhance access to health services, their scalability remains challenging due to multiple systems, as well as regulatory and financial barriers. Despite the demonstrated benefits such as improved patient satisfaction, quality outcomes, and efficiency, expanding these models on a larger scale are often hindered by restrictive scope-of-practice regulations, inadequate funding mechanisms, workforce limitations, and lack of standardized implementation frameworks. According to Ortiz [47], NP owned practices face significant financial and operational challenges that hinder their ability to thrive due to low reimbursement rates. Additionally, opening an NP practice is harder than opening a physician-led practice due to payer misunderstanding of scope of practice and limited access to funding and financing [48].

The future of NMHCs depends on aligning nursing leadership, strengthening workforce development and educational pathways, policy reform, and funding for nurse-led initiatives. APRN education should embed competencies in business operations and policy advocacy to equip APRNs to implement and manage nurse-led models of care. Academic practice partnerships such as UHCON-HC can provide experiential learning for nursing and other healthcare students while delivering community care. These collaborative arrangements fill the gap between education and practice. Future efforts must focus on standardization of practice and policy advocacy for equitable reimbursement for nurse-led services.

Future studies should continue examining the feasibility and acceptability of nurse-led clinics to refine this novel model of care with homeless, working poor, and other underserved populations. Prospective efficacy studies are needed for more definitive conclusions on the impact of nurse-led clinics [35]. Different service configurations and partnership models may impact care outcomes and sustainability, needing further examination. Challenges related to retention deserve further study as well as the impact of services on continuity of care, health, and well-being.

## Figures and Tables

**Table 1:** Characterization of the UHCON-HC patients by demographic characteristics and social determinants of health (*N* = 514).

Characteristic ^a^	*n*	%	*M* (*SD*)	Range (Min/Max)
**Age**			49.03 (13.34)	18.80
**Gender**				
Female	142	28.57		
Male	352	70.82		
Other ^b^	3	0.60		
**Race**				
White	171	37.75		
Black or African American	259	57.17		
Asian	6	1.32		
American Indian or Alaska Native	11	2.43		
Native Hawaiian or Other Pacific Islander	5	1.10		
Arab/Middle Eastern	1	0.22		
**Ethnicity**				
Hispanic or Latino	96	18.68		
Not Hispanic or Latino	347	67.51		
Unknown/Not reported	71	13.81		
**Marital Status**				
Single	324	68.79		
Married/living with partner	37	7.86		
Separated/Divorced	83	17.62		
Widowed	19	4.03		
Other status	8	1.70		
**Education**				
Less than High School	79	16.56		
GED	81	16.98		
High School Diploma	127	26.62		
Some college	113	23.69		
Associate degree	34	7.13		
Bachelor’s degree	36	7.55		
Master’s or Doctorate	7	1.47		
**Employment**				
Unemployed	253	53.60		
Unable to work	108	22.88		
Full employment	32	6.78		
Part-time employment	55	11.65		
Retired	24	5.08		
**Homelessness**				
No	120	26.49		
Yes—sometimes	128	28.26		
Yes—often	34	7.51		
Yes—most of the time	171	37.75		
**Housing**				
Outdoors	103	23.52		
Shelter	125	28.54		
Transitional/reentry housing	34	7.76		
Group home	19	4.34		
Hotel/motel	4	0.91		
Apartment	92	21.00		
House	32	7.31		
Home of friend or family	28	6.39		
Other	1	0.23		
**Access to Care**				
Primary care provider (No)	423	82.30		
Seeing any provider for current health issues (No)	423	82.30		
**Visited the ER recently** (Yes)	168	32.68		
**Any type of health insurance** (No)	311	60.51		
**Transportation** (check all that apply)				
Metro bus	307	59.73		
Metro train	245	47.67		
Own vehicle	49	9.53		
Bicycle	16	3.11		
Friends	22	4.28		
Walk	204	39.69		

^a^ Participants who omitted a question were not included in the denominator when calculating frequencies or percentages for the given characteristic. ^b^ “Other” includes the following: Transgender male/Trans Man/Female-to-male; Transgender woman/Trans Female/Male-to-female; Gender queer, neither exclusively male nor female.

**Table 2:** Feasibility results for services rendered by the UHCON-HC over 3 years, by year, and total.

Services ^a^	Year 1	Year 2	Year 3	Total (3 Years)
	*n*	*n*	*n*	*N*
New Patient Visits	94	163	257	514
Follow-up Visits	87	302	469	858
Total Completed	181	465	726	1372
Seen	181	465	726	1372
Canceled	19	60	49	128
No show	68	166	238	472
Total Scheduled	268	691	1013	1972
Ratio Completed/Scheduled (%)	67.54	67.29	71.67	69.57
Number of Visits per Patient	***M (SD)***	***M (SD)***	***M (SD)***	***M (SD)***
	3.61 (5.36)	3.23 (4.29)	2.31 (2.07)	2.84 (3.67)
Number of Visits per Patient (%)				
1 visit	50.83	37.85	40.36	40.88
2–5 visits	35.92	36.31	43.39	40.11
>5 visits	13.25	25.84	16.25	19.01

^a^ Services operated 1 day per week in Year 1 and 2 days per week in Years 2 and 3.

**Table 3:** Acceptability of the UHCON-HC services: Descriptive statistics of patient satisfaction with facets of care and global satisfaction (PSQ-B) over 3 years.

PSQ-B Facets of Care (Likert Scale 1–5) ^a^	Year 1	Year 2	Year 3	3 Years	3 Years	3 Years
	*M (SD)*	*M (SD)*	*M (SD)*	*M (SD)*	*% Very Satisfied* ^b^	*n*
Access (7 items)	4.69 (0.62)	4.74 (0.60)	4.52 (0.70)	4.63 (0.66)	69.6–75.0	393
Communication/Information (4 items)	4.72 (0.65)	4.77 (0.53)	4.57 (0.69)	4.66 (0.64)	71.5–73.4	387
Interpersonal Relations (6 items)	4.75 (0.59)	4.80 (0.43)	4.59 (0.67)	4.69 (0.59)	74.6–77.0	392
Team Coordination (1 item)	4.75 (0.67)	4.79 (0.50)	4.58 (0.69)	4.68 (0.63)	74.6	374
**PSQ-B Total Scale** (18 items)	4.72 (0.59)	4.76 (0.52)	4.56 (0.68)	4.66 (0.62)	69.6–77.0	396
**PSQ-B Global Satisfaction** (1 item)	4.75 (0.75)	4.82 (0.42)	4.62 (0.66)	4.71 (0.61)	76.3	371

^a^ 1 = Very dissatisfied; 2 = Dissatisfied; 3 = Neither satisfied nor dissatisfied; 4 = Satisfied; 5 = Very satisfied. ^b^ For multi-item scales, range of “%Very satisfied” is reported.

## Data Availability

The data presented in this article are not readily available because the data is part of an ongoing study. The study data will be made available upon reasonable request from the corresponding author.

## Public Involvement Statement

No public involvement in any aspect of this research.

## Guidelines and Standards Statement

This manuscript was drafted against the (STROBE) for a cross-sectional study, descriptive research.

## Abbreviations

The following abbreviations are used in this manuscript:

SDOH	Social Determinants of Health
NMHC	Nurse-managed Health Clinic
APRN	Advanced Practice Registered Nurses
NP	Nurse Practitioner
NASEM	National Academies of Science, Engineering and Medicine
UH	University of Houston
UHCON-HC	University of Houston College of Nursing Health Clinic
NLMC	Nurse-Led Model of Care
PSQ-B	Patient Satisfaction Questionnaire-Brief
SPSS	Statistical Package for the Social Sciences
SAS	Statistical Analyses System
Y1, Y2, Y3	Year 1, Year 2, Year 3
